# Longitudinal and postural changes of blood pressure predict dementia: the Malmö Preventive Project

**DOI:** 10.1007/s10654-017-0228-0

**Published:** 2017-02-11

**Authors:** Hannes Holm, Katarina Nägga, Erik D. Nilsson, Olle Melander, Lennart Minthon, Erasmus Bachus, Artur Fedorowski, Martin Magnusson

**Affiliations:** 1 Department of Clinical Sciences, Clinical Research Center, Lund University, Malmö, Sweden; 2 Department of Internal Medicine, Skåne University Hospital, Jan Waldenströms gata 35, 205 02 Malmö, Sweden; 3 Clinical Memory Research Unit, Department of Clinical Sciences Malmö, Lund University, Malmö, Sweden; 4 Department of Cardiology, Skåne University Hospital, Malmö, Sweden

**Keywords:** Dementia, Orthostatic hypotension, Blood pressure, Prospective studies, Risk factors

## Abstract

The role of blood pressure (BP) changes in dementia is debatable. We aimed to analyse how resting and postural BP changes relate to incident dementia over a long-term follow-up. In the prospective population-based Malmö Preventive Project, 18,240 study participants (mean age: 45 ± 7 years, 63% male) were examined between 1974 and 1992 with resting and standing BP measurement, and re-examined between 2002 and 2006 at mean age of 68 ± 6 years with resting BP. A total of 428 participants (2.3%) were diagnosed with dementia through Dec 31, 2009. The association of resting and postural BP changes with risk of dementia was studied using multivariable-adjusted Cox regression models controlling for traditional risk factors. Diastolic BP (DBP) decrease on standing indicated higher risk of dementia [Hazard ratio (HR) per 10 mmHg: 1.22; 95% confidence interval (CI) 1.01–1.44, *p* = 0.036], which was mainly driven by increased risk in normotensive individuals. Higher systolic (SBP) and diastolic BP at re-examination was associated with lower risk of dementia (HR per 10 mmHg: 0.94; 95% CI 0.89–0.99, *p* = 0.011; and 0.87; 0.78–0.96, *p* = 0.006, respectively). Extreme decrease in SBP/DBP between baseline and re-examination (4th quartile; −7 ± 12/−15 ± 7 mmHg, respectively) indicated higher risk of dementia (HR 1.46; 95% CI 1.11–1.93, *p* = 0.008, and 1.54; 95% CI 1.14–2.08, *p* = 0.005; respectively) compared with reference group characterised by pronounced BP increase over the same period (1st quartile; +44 ± 13/+15 ± 7 mmHg). Diastolic BP decrease on standing in the middle age, decline in BP between middle-and advanced age, and lower BP in advanced age are independent risk factors of developing dementia.

**Electronic supplementary material:**

The online version of this article (doi:10.1007/s10654-017-0228-0) contains supplementary material, which is available to authorized users.

## Introduction

Dementia, an escalating health issue with the advancing age in industrialized countries, is a collective term for brain disorders associated with failure of cognitive functions including memory, mental speed, executive functions and speech [1]. Previous studies have reported a possible association between high BP levels in midlife and development of dementia later in life [2, 3]. In parallel, it has been demonstrated that both orthostatic hypotension (OH), a manifestation of autonomic failure, and symptoms of orthostatic intolerance in personal history such as light-headedness and dim vision may predict mild cognitive decline [4]. However, OH is often asymptomatic i.e. without clinical signs indicating an underlying disorder, thus making the patient unaware of the problem. OH is a common condition among older individuals with a reported prevalence between 5 and 30% [5]. In population-based prospective studies OH has been consistently linked with increased mortality and cardiovascular (CV) morbidity [6, 7] but data on association between OH and dementia are very sparse. Impairment of orthostatic BP response is often associated with elevated BP and antihypertensive treatment [7] while low habitual BP increases the risk of cerebral hypoperfusion in patients with OH, although cerebral autoregulation may effectively prevent symptoms [8]. However, most studies regarding the association between impaired BP control and dementia are cross-sectional, describing the coexistence of these conditions at a specific point of time [9]. Thus, there is a need for prospective studies investigating the role of BP changes, including OH, in developing dementia. To this end, we assessed the longitudinal relationship between resting BP, postural BP response and incident dementia in a large population-based cohort with a long-term follow-up.

## Methods

### Study population

**Fig. 1:**
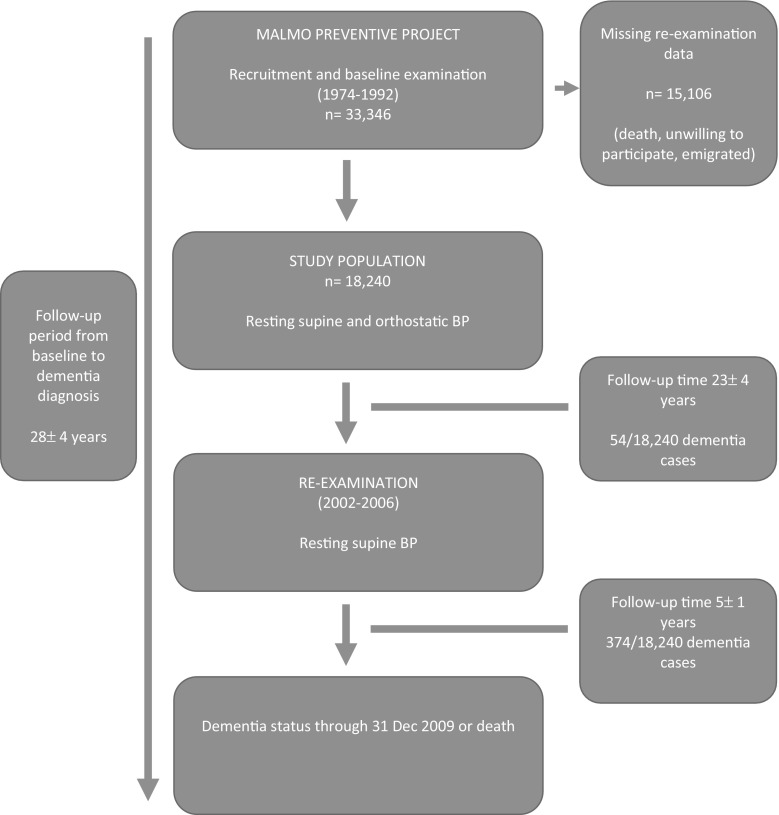
Malmö Preventive Project and re-screening program

The Malmö Preventive Project (MPP) was funded in the mid 1970s at the Malmo University Hospital in purpose to explore CV risk factors. Between 1974 and 1992, a total of 33,346 individuals living in Malmo were included. At baseline, participants were screened for hypertension, diabetes, obesity, hyperlipidaemia, smoking, family history of CV disease and other potential CV risk factors. A detailed description of baseline examination has been published elsewhere [7]. Between 2002 and 2006, a total of 18,240 of the surviving individuals were re-examined constituting the present study population (Fig. 1). All participants who attended the rescreening program gave an informed consent and were thus eligible for the study of dementia [10].

### Blood pressure measurements at baseline and re-examination

Trained nurses measured blood pressure (BP) using an auscultatory method with a mercury sphygmomanometer and an appropriately sized cuff placed around the right arm supported at the heart level [7]. The first BP reading was assessed twice after 10 min of rest in supine position. Then, the participants were asked to stand up and the second BP measurement was taken twice after 1 min in the standing position. All values were rounded up to the nearest 5 mmHg and recorded in the database, and the mean value of the two measurements in each position was calculated. During re-examination, which was performed according to the same protocol as baseline screening, only supine BP was measured [10]. A validated automated sphygmomanometer was used instead of manual measurements and values were rounded up to the nearest integer.

### Dementia diagnosis

Information about dementia diagnosis was requested from the Swedish National Patient Register (SNPR) and covered the period from baseline through Dec 31, 2009. The diagnoses in the register were coded according to the International Classification of Diseases (ICD 8th, 9th, and 10th revisions). Since 1987, SNPR includes all in-patient care in Sweden and, in addition, contains data on outpatient visits including day surgery and psychiatric care from both private and public caregivers recorded not earlier than in 2001. Of note, primary care is not yet covered in the SNPR. Dementia diagnoses were validated by a thorough review of medical records as well as neuroimaging data when available. A research physician assigned the final diagnosis for each patient and a geriatrician specialized in cognitive disorders was consulted in unclear cases. All-cause dementia was diagnosed according to the criteria of the Diagnostic and Statistical Manual of Mental Disorders, 3rd edition, revised (DSM-IIIR). For retrieving the diagnoses of AD and VaD, the DSM-IV criteria were used. When the clinical and cognitive presentation and neuroimaging findings all were consistent with either of these diagnostic categories, the diagnoses were set. In cases where the clinical presentation and findings on neuroimaging implied both AD and VaD, the diagnosis of mixed dementia was established. 471 individuals had a dementia diagnose registered in SNPR. Of these, 428 cases were classified as validated dementia diagnoses including; 142 Alzheimer’s disease, 96 Vascular dementia, 114 Mixed type, 38 Lewy-body-dementia/Parkinson dementia, 4 fronto-temporal dementia, 34 unspecified type. Of the 428 validated diagnoses, 54 individuals were diagnosed with dementia before the re-examination in MPP, and 374 individuals were diagnosed between the re-examination and Dec 31, 2009.

### Statistical methods

Group differences in continuous variables between dementia-positive and -negative individuals were compared using One-Way ANOVA test, whereas categorical variables were compared using Pearson’s Chi-square test. The longitudinal associations of incident dementia with BP recorded during baseline and rescreening examinations, including data on postural i.e. orthostatic systolic (SBP) and diastolic (DBP) BP reaction at baseline were studied. The categorical OH variable of standing SBP decrease ≥20 mmHg and/or DBP decrease ≥10 mmHg [11] demonstrated a very low prevalence (383/17,493; 2.1%) in the re-examined subset compared with the original MPP cohort (6.1%) [7]. Cox regression model was applied entering supine SBP/DBP at baseline or re-examination, categorical OH, orthostatic SBP/DBP reaction at baseline, difference in SBP/DBP between baseline and re-examination, and prevalent hypertension defined as SBP > 140 mmHg or DBP > 90 mmHg or self-reported antihypertensive treatment as independent variables. The adjusted model was built by entering age, gender, antihypertensive treatment, diabetes, smoking, prevalent CV disease, and plasma-cholesterol as covariates. Further, BP-related variables (orthostatic SBP/DBP reaction, supine SBP/DBP at re-examination, and SBP/DBP difference between baseline and re-examination) were stratified into quartiles and used for Kaplan–Meier survival analysis, and as an independent variable for Cox regression analysis in order to test the risk increment across the quartiles of BP-derived parameters. The time variable was calculated as follow-up time between baseline or rescreening, respectively, and date of dementia diagnosis, death, or end of follow-up on Dec 31, 2009. In the rescreening-to-dementia risk analyses, 54 participants with prevalent dementia diagnosis at rescreening were excluded. The missing data ranged from 3 to 365 cases for different variables, and the cases were not included in the respective analyses. All analyses were performed using IBM SPSS Statistics version 22 (SPSS Inc., Chicago, IL, USA). All tests were two-sided, whereby *p* < 0.05 was considered statistically significant.

## Results

At baseline, study participants that developed dementia during follow up period (n = 428) were older, more likely to be women and had higher probability of diabetes (see Table 1). Participants that were diagnosed with dementia had higher supine SBP and DBP compared to the rest of cohort, and demonstrated more pronounced SPB fall and less pronounced DBP increase on standing (Table 1). The mean follow-up period from baseline to rescreening was 23 ± 4 years, and the total duration of follow-up period from baseline to dementia diagnosis or end of follow-up was 28 ± 4 years (Fig. 1). At rescreening, dementia participants were older and had lower supine SBP and DBP compared with individuals free from dementia, while proportion of antihypertensive treatment was slightly higher in dementia-positive group (43 vs. 38%, *p* = 0.057) (Table 1).

**Table 1:** Characteristics of study participants (n = 18,240) at baseline and reexamination stratified according to dementia diagnosis during follow-up period

Characteristic	Dementia positive	Dementia negative	*p* value
	n = 428	n = 17,812	
*Baseline*			
Age (years)	50 ± 5	45 ± 7	<0.001
Sex [% (male)]	59	64	<0.001
Current smoker (%)	40	37	0.2
BMI (kg/m^2^)	25 ± 3	24 ± 3	0.91
Supine systolic BP (mmHg)	131 ± 16	127 ± 14	<0.001
Supine diastolic BP (mmHg)	86 ± 10	85 ± 9	<0.001
Antihypertensive treatment [% (n)]	7	4	0.003
Hypertension	44.1	34.4	<0.001
Orthostatic systolic BP reaction (mmHg)	−2.8 ± 7	−1.4 ± 7	<0.001
Orthostatic diastolic BP reaction (mmHg)	+1.7 ± 5	+2.5 ± 5	<0.001
Diabetes (%)	4.4	3.1 (559)	0.018
Plasma cholesterol (mmol/l)	6.0 ± 1	5.5 ± 1	<0.001
*Reexamination*			
Age (years)	73 ± 5	68 ± 6	<0.001
Current smoker (%)	14	14	1.0
Systolic BP (mmHg)	143 ± 21	145 ± 20	0.034
Diastolic BP (mmHg)	81 ± 11	84 ± 11	<0.001
Antihypertensive treatment (%)	43	38	0.057
Hypertension (%)	71.9	72.2	0.9
Diabetes (%)	20	11	0.18
Plasma cholesterol (mmol/l)	5.7 ± 1.1	5.6 ± 1.1	0.62

Values are displayed as mean ± SD or frequency in percent

*BMI* body mass index, *BP* blood pressure

### Postural BP decrease and supine BP at baseline versus dementia

In the multivariable Cox regression model (Table 2), postural DBP decrease, but not SBP decrease at baseline was significantly associated with the risk of developing dementia [Hazard ratio (HR) per 10 mmHg: 1.22; 95% confidence interval (CI) 1.01–1.44, *p* = 0.036, and 1.02; 0.89–1.15, *p* = 0.74, respectively]. As can be seen in Table 3, a distinct postural DBP decrease (4th quartile; −4 ± 3 mmHg) indicated increased dementia risk (HR 1.41; 95% CI 1.02–1.94, *p* = 0.036) compared with reference group (1st quartile; +9 ± 3 mmHg), which did not substantially differ in dementia risk from the rest of cohort (2nd and 3rd quartiles). Further adjustment for body-mass index, socioeconomic status (lower-higher), and alcohol consumption (overconsumption–normal consumption) did not significantly change our results (data not shown). The risk of dementia associated with postural DBP decrease was more pronounced for vascular type compared with combined Alzheimer’s disease and mixed type (see Table 4). Ancillary analysis showed that postural DBP decrease was not associated with combined endpoint of incident dementia or death (Supplementary Table 1).

**Table 2:** Relationship between blood pressure levels at baseline and re-examination and dementia risk

Characteristic	HR, 95% CI^a^ (per 10 mmHg)	*p* value
Baseline supine SBP (n = 17,912)	1.04 (0.98–1.10)	0.19
Baseline supine DBP (n = 17,909)	1.05 (0.95–1.16)	0.30
Orthostatic SBP reaction (n = 17,884)	1.02 (0.89–1.15)	0.74
Orthostatic DBP reaction (n = 17,875)	1.22 (1.01–1.44)	0.036
Orthostatic hypotension^b^ (383/17,492)	1.18 (0.73–1.89)	0.51
Re-examination SBP (n = 18,044)	0.94 (0.89–0.99)	0.011
Re-examination DBP (n = 18,043)	0.87 (0.78–0.96)	0.006
SBP decrease between baseline and re-examination (n = 17,719)	1.07 (1.03–1.12)	0.002
DBP decrease between baseline and re-examination (n = 17,715)	1.16 (1.08–1.25)	<0.001

*HR* hazard ratio, *CI* confidence interval, *SBP* systolic blood pressure, *DBP* diastolic blood pressure

^a^Adjusted for age, gender, anti-hypertensive treatment, smoking, diabetes, prevalent cardiovascular disease, and plasma-cholesterol

^b^Orthostatic hypotension is a categorical variable

**Table 3:** Associations between dementia and blood pressure variations across quartiles of blood pressure-derived parameters

Quartiles	n	HR, 95% CI^a^	*p* value
*Orthostatic DBP reaction (baseline)*			
Q1 (≥7.5 mmHg)	3161	Reference	
Q2 (2.5–5.0 mmHg)	6995	1.06 (0.79–1.43)	0.68
Q3 (0 mmHg)	4834	1.03 (0.75–1.41)	0.86
Q4 (≤−2.5 mmHg)	2885	1.41 (1.02–1.94)	0.036
*p* for trend		0.072	
*SBP at re*-*examination*			
Q1 (≥158 mmHg)	4466	Reference	
Q2 (143–157 mmHg)	4638	1.16 (0.87–1.54)	0.31
Q3 (131–143 mmHg)	4480	1.11 (0.83–1.45)	0.49
Q4 (≤130 mmHg)	4444	1.48 (1.12–1.94)	0.006
*p* for trend		0.032	
*DBP at re*-*examination*			
Q1 (≥91 mmHg)	4480	Reference	
Q2 (83–90 mmHg)	4466	1.10 (0.81–1.49)	0.53
Q3 (77–83 mmHg)	4632	1.15 (0.85–1.55)	0.37
Q4 (≤76 mmHg)	4449	1.33 (1.00–1.78)	0.050
*p* for trend		0.24	
*Difference in SBP between re*-*examination and baseline*			
Q1 (≥30 mmHg)	4483	Reference	
Q2 (17–29 mmHg)	4338	1.05 (0.77–1.42)	0.77
Q3 (5–16 mmHg)	4455	1.15 (0.85–1.55)	0.36
Q4 (≤4 mmHg)	4427	1.46 (1.1–1.93)	0.008
*p* for trend		0.023	
*Difference in DBP between re*-*examination and baseline*			
Q1 (≥8 mmHg)	4378	Reference	
Q2 (0–7 mmHg)	4527	1.19 (0.86–1.65)	0.29
Q3 (−1 to −7 mmHg)	4297	1.18 (0.85–1.63)	0.31
Q4 (≤−8 mmHg)	4497	1.54 (1.14–2.08)	0.005
*p* for trend		0.024	

*HR* hazard ratio, *CI* confidence interval, *SBP* systolic blood pressure, *DBP* diastolic blood pressure

^a^Adjusted for age, gender, anti-hypertensive treatment, smoking, diabetes, prevalent cardiovascular disease, and plasma-cholesterol

**Table 4:** Relationship between blood pressure levels at baseline and re-examination and subtypes of dementia

Characteristic	HR, 95% CI^a^ (per 10 mmHg)	*p* value	HR, 95% CI^a^ (per 10 mmHg)	*p* value
	AD + Mixed Type (n = 156)		Vascular (n = 96)	
Baseline supine SBP	0.98 (0.90–1.06)	0.62	1.23 (1.12–1.35)	<0.001
Baseline supine DBP	0.96 (0.82–1.10)	0.55	1.48 (1.27–1.68)	<0.001
Orthostatic SBP reaction	0.97 (0.80–1.13)	0.70	1.24 (0.99–1.50)	0.064
Orthostatic DBP reaction	1.16 (0.89–1.43)	0.26	1.46 (1.03–1.90)	0.035
Orthostatic hypotension^b^	0.96 (0.49–1.88)	0.91	1.99 (0.91–4.35)	0.086
Re-examination SBP	0.95 (0.98–1.01)	0.091	0.95 (0.85–1.06)	0.383
Re-examination DBP	0.87 (0.75–0.99)	0.031	0.91 (0.71–1.11)	0.377
SBP decrease between baseline and re-examination	1.06 (1.01–1.12)	0.023	1.17 (1.09–1.26)	<0.001
DBP decrease between baseline and re-examination	1.06 (0.96–1.16	0.236	1.33 (1.19–1.48)	<0.001

*AD* Alzheimer’s disease, *SBP* systolic blood pressure, *DBP* diastolic blood pressure

^a^Adjusted for age, gender, anti-hypertensive treatment, smoking, diabetes, prevalent cardiovascular disease, and plasma-cholesterol

^b^Orthostatic hypotension is a categorical variable

Supine SBP and DBP at baseline were not associated with increased incidence of all-cause dementia, neither as a continuous variable (HR per 10 mmHg: 1.04; 95% CI 0.98–1.10, *p* = 0.19, and 1.05; 95% CI 0.95–1.16, *p* = 0.30, respectively) nor in quartile and age- and gender-stratified analyses (data not shown). However, vascular type of dementia showed strong association with elevated BP at baseline (see Table 4).

### Supine BP at re-examination versus dementia

At re-examination, higher systolic and diastolic BP values were associated with lower risk of dementia (HR per 10 mmHg: 0.94; 95% CI 0.89–0.99, *p* = 0.011; and 0.87; 0.78–0.96, *p* = 0.006, respectively; Table 2). As shown in Table 3, lowest SBP (4th quartile; 121 ± 8 mmHg) was associated with increased dementia risk (HR 1.48; 95% CI 1.12–1.94, *p* = 0.006) compared with highest SBP (reference group, 1st quartile; 172 ± 13 mmHg). Similarly, lowest DBP (4th quartile; 71 ± 5 mmHg) was associated with increased dementia risk (HR 1.33; 95% CI 1.00–1.78, *p* = 0.05) compared with highest DBP (reference group; 1st quartile; 98 ± 6 mmHg).

### Longitudinal changes in BP versus dementia

**Fig. 2:**
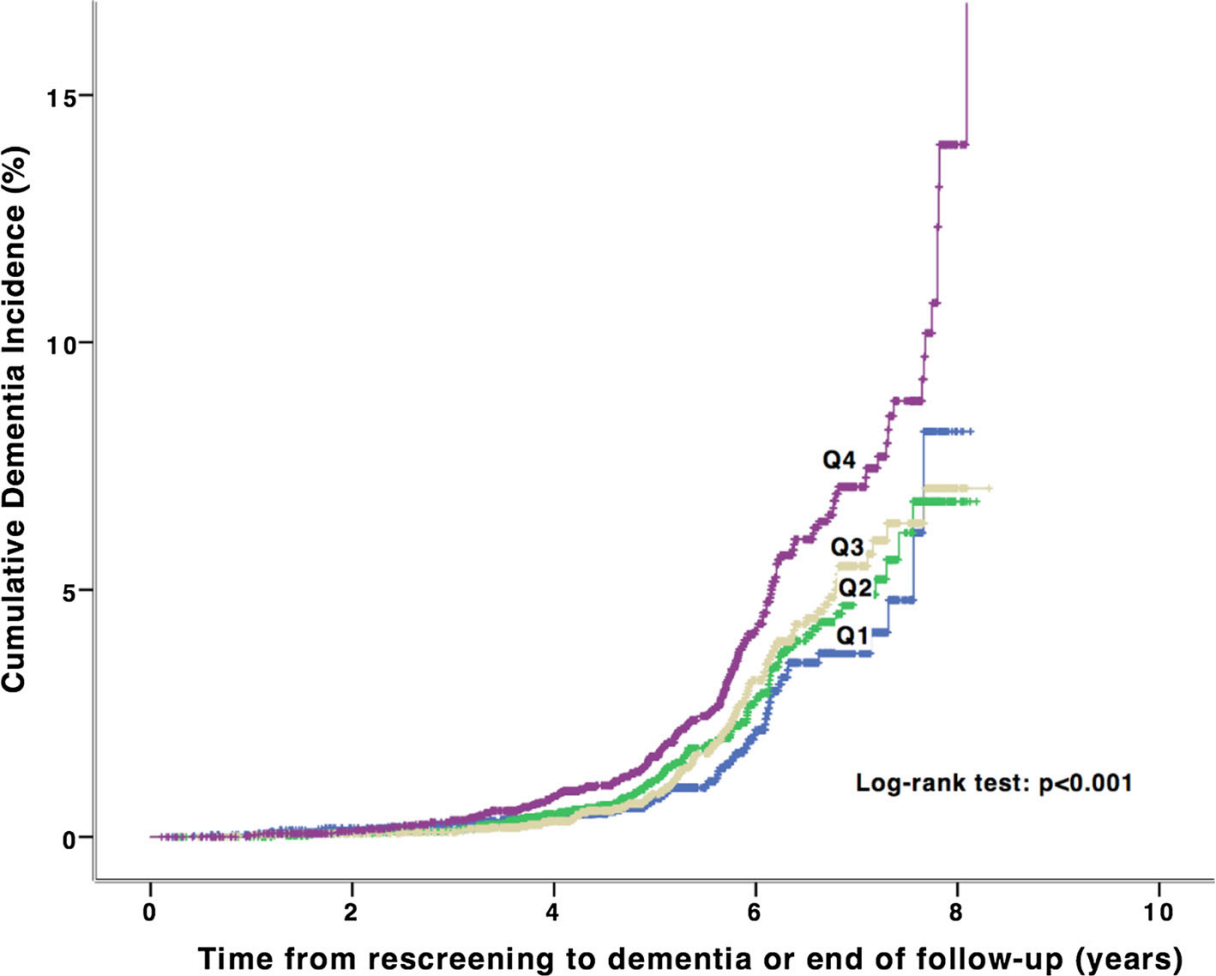
Kaplan–Meier curves for cumulative dementia incidence (n = 374) from rescreening (2002–2006) to the end of follow-up (Dec 31, 2009) among 18,240 participants of Malmö Preventive Project stratified according to quartiles of systolic blood pressure change between baseline (1974–1992) and rescreening. Q1 44 ± 13 mmHg; Q2 23 ± 7 mmHg; Q3 11 ± 4 mmHg; Q4 −7 ± 12 mmHg

**Fig. 3:**
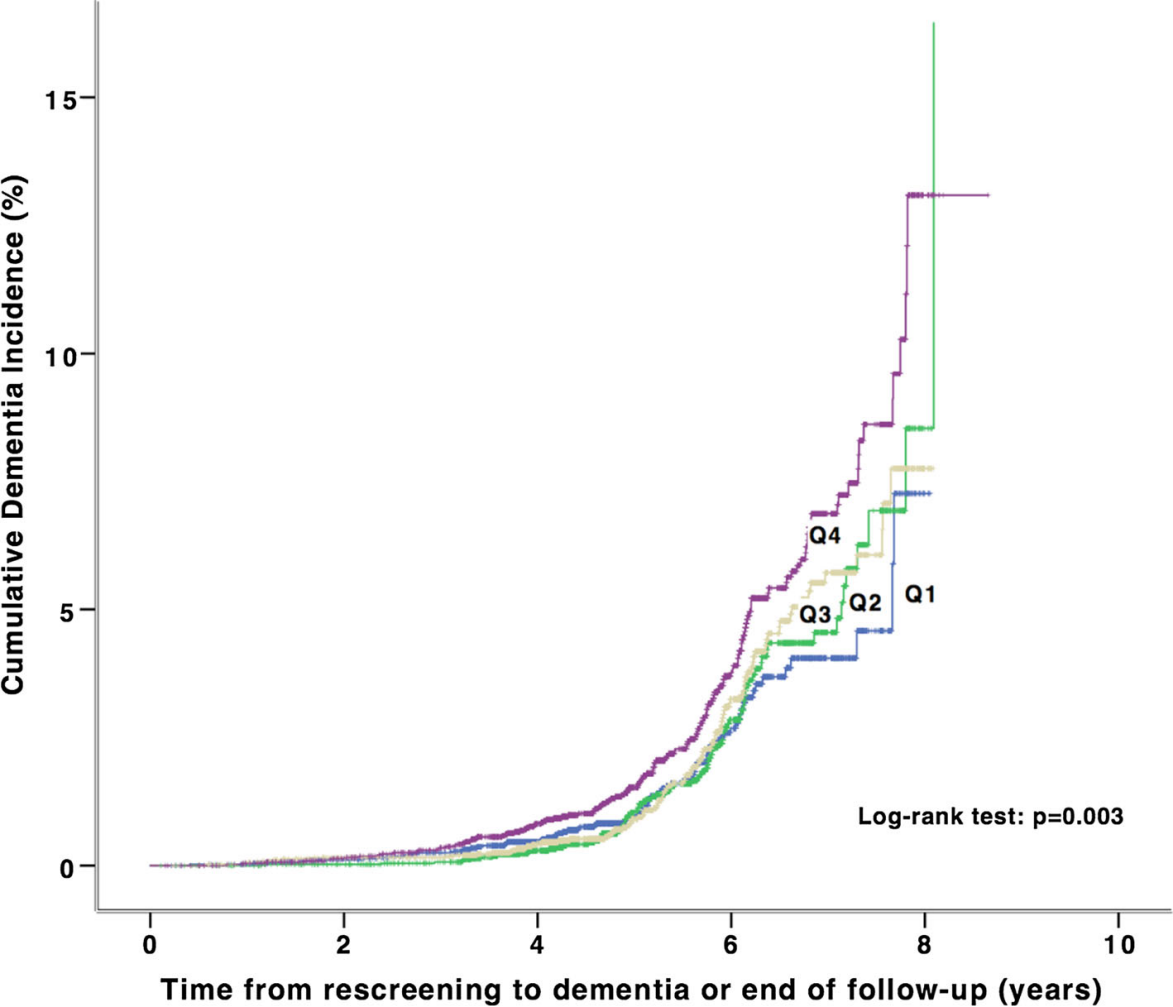
Kaplan–Meier curves for cumulative dementia incidence (n = 374) from rescreening (2002–2006) to the end of follow-up (Dec 31, 2009) among 18,240 participants of Malmö Preventive Project stratified according to quartiles of diastolic blood pressure change between baseline (1974–1992) and rescreening. Q1 +15 ± 7 mmHg; Q2 3 ± 2 mmHg; Q3 −4 ± 2 mmHg; Q4 −15 ± 7 mmHg

SBP decrease between baseline and re-examination was associated with increased risk of dementia (HR 1.07; 95% CI 1.03–1.12, *p* = 0.002) and so was DBP decrease between baseline and re-examination (HR 1.16; 95% CI 1.08–1.25, *p* < 0.001; Table 2). Quartile analysis (see Fig. 2; Table 3) revealed that SBP decrease between baseline and re-examination (4th quartile; −7 ± 12 mmHg) was associated with increased risk of dementia (HR 1.46; 95% CI 1.11–1.93, *p* = 0.008) compared with reference group (1st quartile; +44 ± 13 mmHg). Analogically, pronounced DBP decrease between baseline and re-examination (4th quartile; −15 ± 7 mmHg) indicated increased risk of dementia (HR 1.54; 95% CI 1.14–2.08, *p* = 0.005) compared with reference group (1st quartile; +15 ± 7 mmHg). For all analyses, further adjustments for body-mass index, socioeconomic status (lower-higher), and alcohol consumption (overconsumption–normal consumption) at baseline and also incident diabetes during follow-up period did not significantly change the results (Fig. 3).

### Hypertension and antihypertensive treatment versus dementia

Hypertension was more prevalent at re-examination compared with baseline (72 vs. 34%; Table 1). There was no effect of hypertension/antihypertensive treatment at either baseline or re-examination on the development of dementia (data not shown). We found one significant interaction, between hypertension at baseline and orthostatic DBP reaction (*p* = 0.001). Postural DBP decrease was associated with higher risk of dementia in normotensive (HR 1.51; 95% CI 1.21–1.81, *p* = 0.001) but not in hypertensive individuals (HR 0.87; 95% CI 0.57–1.19, *p* = 0.43).

## Discussion

In this study, we have found that decrease in diastolic blood pressure on standing in the middle age, decline in blood pressure between middle-and advanced age, and lower blood pressure in the advanced age are all independent risk factors of developing dementia. The risk of dementia was highest in the extreme quartiles of assessed BP-related parameters: the most pronounced DBP fall on standing, lowest SBP and DBP at re-examination, and progression from higher to lower SBP and DBP between baseline and re-examination. Moreover, postural BP decrease indicated increased risk of dementia development in normotensive individuals only. Finally, higher BP at baseline was predictive of vascular dementia but not Alzheimer’s disease and mixed type.

In our cohort, the prevalence of OH according to consensus criteria was very low (≈2%), most likely due to the fact that patients with OH exceeded in mortality the rest of baseline population [12] and, consequently, were not included in re-examination. Thus, apart from categorical OH, we decided to study postural i.e. orthostatic BP changes as a continuous variable. On the other hand, most of literature on dementia and orthostatic impairment report on categorical OH but longitudinal studies are very sparse [13].

In order to understand a possible relationship between orthostatic BP fall and cognitive decline, cerebral perfusion and blood flow have been studied. Using electroencephalography it has been demonstrated that cerebral blood flow is reduced in OH, which may lead to cerebral damage and cognitive impairment [14]. Additionally, single-photon emission computed tomography of the human brain has shown that during orthostatic stress the brain perfusion is reduced [15]. The reduced cerebral blood flow seen in participants with orthostatic intolerance may result from dysfunction of the cerebral autoregulation, which plays an important role in order to maintain adequate cerebral blood flow and pressure [16]. The cerebral perfusion pressure is preserved between ~60 and 160 mmHg of systemic SBP [17]. Below 60 mmHg, the cerebral autoregulation collapses and the reduction of blood flow is compensated for by enhanced oxygen extraction [18]. If the improved oxygen extraction fails to deliver enough amount of oxygen to cerebral tissues, cerebral hypoxia ensues which may result in irreversible tissue damage and development of dementia. Given that the prevalence of orthostatic and non-orthostatic hypotension reached 50% in clinically evaluated vascular dementia cases [19], one possible explanation why OH individuals have higher burden of dementia might be BP fall below limits of autoregulation during orthostatic challenge. In addition, it has previously been indicated that patients with autonomic dysfunction have more severe neuropsychological deficits [20]. This might be a consequence of cholinergic dysfunction, which may be a possible cause of autonomic failure in patients with dementia. Therefore, in further studies it will be important to examine how cholinesterase inhibitor therapy affects dementia patients with autonomic dysfunction.

The postural DBP decrease predicted dementia development in normotensive individuals only. A possible explanation is that these individuals already have lower BP and therefore are more sensitive to BP drop, which might significantly reduce cerebral perfusion if the critical level is reached. Orthostatic hypotension has been associated with arterial stiffness [21] and earlier studies have implicated association between arterial stiffness and higher prevalence of cognitive dysfunction [22]. Consequently, reduced arterial compliance might mediate the increased risk of dementia seen in participants with lower BP and postural DBP decline. Further, with advancing age the small resistance blood vessels undergo degenerative changes consisting of thickening and fibrosis of the media and intima, and patchy degeneration of smooth muscle cells producing luminal narrowing and increased vascular resistance. Although the resting cerebral blood flow is the same in normotensive and hypertensive individuals, these structural changes limit the capacity of the resistance vessels for maximal vasodilatation and impair tolerance of lower BP. When abrupt changes in BP occur, the cerebral autoregulation does not have the ability of BP adaptation, which may result in disruptions in neurovascular coupling and neurodegenerative changes [23]. Reduction of both SBP and DBP has been associated with dementia risk in a number of previous studies [24, 25]. So, while low BP has CVD-protective effects in healthy older individuals, it may constitute a risk factor for hemodynamic instability and cerebral hypoperfusion in the vulnerable older patient [26]. Excessive SBP lowering may therefore be harmful for older patients with cognitive impairment, and BP monitoring can be useful to help avoid BP overtreatment in this population [27].

We observed that study participants who developed dementia demonstrated a bimodal pattern of BP level, higher in the middle age and lower in later life. It supports the hypothesis that low pressure might be a consequence of incipient dementia or, alternatively, a predisposing factor for dementia development. This pattern of BP reduction within the years before clinical signs of dementia appear has been described earlier [2, 28]. Several brain regions affected by dementia progress are involved in BP regulation. They include hypothalamus, amygdala, paraventricular cortex, insular cortex, anterior cingulated cortex, nucleus tractus solitarius, ambiguous nucleus, ventrolateral medulla and tracts in the spinal cord [29]. A reversed causation has been suggested where brain lesions caused by dementia progress result in hypotension. Burke and colleagues reported a strong correlation between decrease in number of C1 neurones in the medulla oblongata and BP dysregulation in Alzheimer patients [30]. High midlife BP has previously been described as an independent risk factor for dementia development [31, 32]. A possible explanation is that hypertension contributes to stroke risk [33] and progress of arteriosclerosis [34], which may also lead to dementia. Neuroimaging and autopsy of participants in the Honolulu-Asia aging study has revealed that individuals with hypertension in midlife have higher incidence of hippocampal atrophy, neurofibrillary tangles and neuritic plaques, often seen in dementia [35]. Our findings emphasises the importance of maintaining a sufficient BP level to preserve cerebral perfusion and thereby cognitive function.

### Strengths and limitations

An important strength of the current study is the use of a well-characterized prospective cohort that has been followed longitudinally for decades. On the other hand, we missed subjects who participated in the MPP baseline exam but died during follow-up or did not participate in the re- examination for other reasons, which could lead to either over- or underestimation of the orthostatic BP change effect on dementia development. However, since OH-positive individuals demonstrated increased mortality compared with the rest of cohort, an underestimation of such associations is rather to be expected. Further, echocardiographic data and information on newly introduced antihypertensive treatment and incident diabetes between rescreening and end of follow up was not available, which might have influenced the results. Since primary care is not covered in the SNPR, an underestimation of dementia cases is possible. Finally, samples were comprised predominantly of individuals of European ancestry, and therefore, the results of this study may not be generalizable to other racial/ethnic groups.

## Conclusion

Diastolic BP fall on standing in the middle age, decrease in BP between middle-and advanced age, and lower BP in the advanced age are independent risk factors of developing dementia. These results support the importance of BP monitoring, including orthostatic test, for prediction of dementia in the population.

## Electronic supplementary material

Below is the link to the electronic supplementary material.

Supplementary material 1 (DOCX 69 kb)
