# Supplementary material for: Longitudinal and postural changes of blood pressure predict dementia: the Malmö Preventive Project
Source: Eur J Epidemiol. 2017 Feb 11;32(4):327–36. doi: 10.1007/s10654-017-0228-0 (PMC5437180; doi:10.1007/s10654-017-0228-0)
Supplement: Supplementary file 1 — Supplementary material 1 (DOCX 69 kb) [file 10654_2017_228_MOESM1_ESM.docx]

**Supplementary Table 1. Relationship between blood pressure levels at baseline and re-examination with combined endpoint of incident dementia or death (n=871) in the multivariable-adjusted Cox regression model.**

| Characteristic | Hazard ratio, 95 % CI *  (per 10 mmHg) | *p-value* |
| --- | --- | --- |
|  |  |  |
|  |  |  |
| Baseline supine SBP | 1.02 (0.98-1.07) | 0.33 |
| Baseline supine DBP | 1.04 (0.96-1.12) | 0.37 |
| Orthostatic SBP reaction | 1.04 (0.95-1.14) | 0.38 |
| Orthostatic DBP reaction | 1.12 (0.96-1.27) | 0.14 |
| Re-examination SBP | 0.95 (0.91-0.98) | 0.004 |
| Re-examination DBP | 0.92 (0.85-0.99) | 0.022 |
| SBP decrease between baseline and re-examination | 1.07 (1.04-1.10) | <0.001 |
| \| DBP decrease between baseline and re-examination \| 1.008 (1.002-1.013) \| 0.001 \| \| --- \| --- \| --- \| | 1.08 (1.02-1.13) | 0.001 |

SBP, systolic blood pressure; DBP, diastolic blood pressure.
